# Harlequin Ichthyosis: A Case Report

**DOI:** 10.1002/ccr3.70623

**Published:** 2025-07-14

**Authors:** Shoaib Akhtar, Adeel Anwaar, Inam Ul Haq, Hazaq Mukhtar, Sabir Jamal, Muhammad Muzammil, Aymar Akilimali

**Affiliations:** ^1^ Rahbar Medical and Dental College Lahore Pakistan; ^2^ Pakistan Kidney and Liver Institute and Research Center Lahore Pakistan Lahore Pakistan; ^3^ Rashid Latif Medical and Dental College Lahore Pakistan Lahore Pakistan; ^4^ Department of Research Medical Research Circle (MedReC) Goma Democratic Republic of Congo

**Keywords:** ABCA12 gene, case report, harlequin ichthyosis

## Abstract

Harlequin ichthyosis (HI) is a genetic disorder caused by ABCA12 gene mutations, presenting with thick, scaly skin and deep fissures. Early recognition, intensive neonatal care, and multidisciplinary management are crucial for improving survival and quality of life. Treatment focuses on skin hydration, infection prevention, and supportive care to manage symptoms effectively.

## 1 Introduction

Harlequin ichthyosis (HI) is one of the most severe forms of congenital ichthyosis, a group of disorders characterized by abnormal skin shedding and keratinization. It is an autosomal recessive condition resulting from mutations in the ABCA12 gene [1, 2, 3], which encodes a transporter protein responsible for lipid transport in the epidermis [4]. This disruption leads to the accumulation of thick scales and the formation of deep, painful fissures in the skin, often leading to systemic complications such as dehydration, electrolyte imbalances, and infections. The classic “harlequin” appearance of the skin—marked by large, diamond‐shaped scales—gives the disorder its name. While the condition was once associated with a high mortality rate, advances in neonatal care and treatment strategies have improved the prognosis for affected individuals. We report a rare case of HI.

## 2 Case Presentation

### 2.1 Case History/Examination

A 21‐year‐old pregnant woman was admitted to Punjab Rangers Teaching Hospital, Lahore, for her second pregnancy due to preterm labor and lower abdominal pain (obstetric pain). The gestational age was approximately 32 weeks based on both the first day of her last menstrual period and ultrasound findings.

### 2.2 Imaging Investigation Findings

Polyhydramnios was observed on ultrasound, with an amniotic fluid index of 26, indicating an excessive accumulation of amniotic fluid. The underlying cause of the condition remained uncertain, as no further diagnostic evaluations had been conducted. Notably, an anomaly scan had not been performed, leaving potential fetal abnormalities unassessed. Further investigations were needed to determine any underlying maternal or fetal factors contributing to the increased fluid levels.

### 2.3 Surgical Management

**FIGURE 1:**
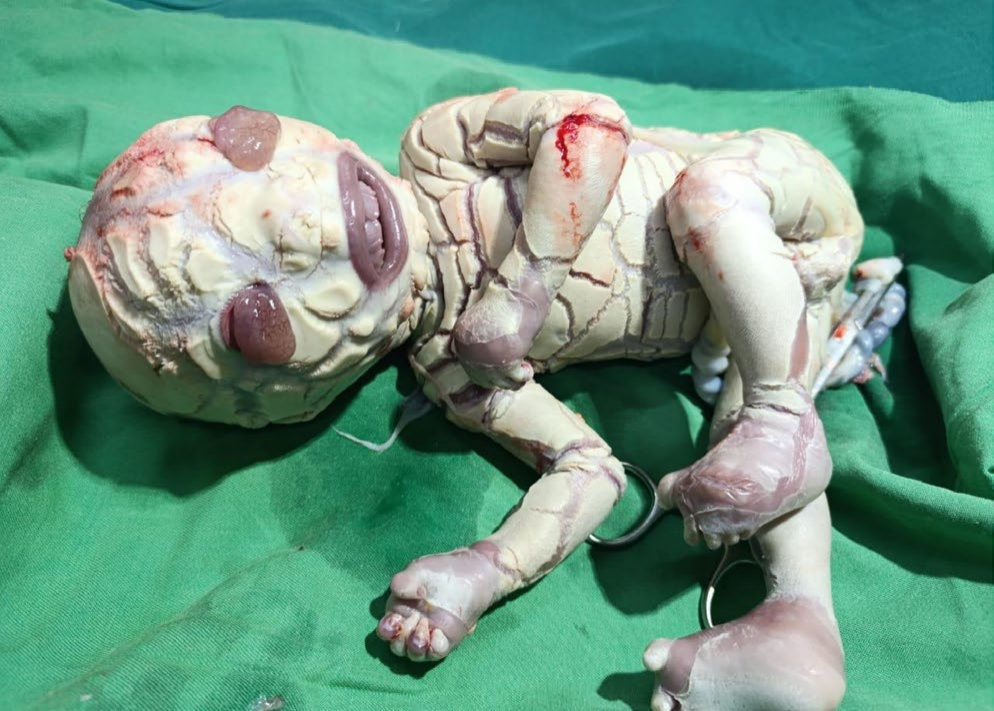
A baby boy with deep cracked skin, a wide‐open mouth, abnormal eyes, and a flattened nose and ear.

A male baby with HI was delivered via lower‐segment cesarean section, highlighting the complexities involved in his perinatal management. His birth weight, occipitofrontal circumference, and length were recorded as 1.56 kg, 31 cm, and 44 cm, respectively, and these measurements indicated that he was significantly small, underscoring his fragile condition. Notably, the physical examination revealed features such as thick skin with deep fissures, generalized hyperkeratinization, cyanosis, flat fontanels, ectropion, immature eyes and auricles, eclabium, and moaning (Figure 1), which collectively emphasized the severity of his congenital condition, while the fact that his parents, who were distantly related, already had one healthy child added an intriguing aspect to his genetic background.

### 2.4 Ongoing Follow‐Up Plan

Antibiotic therapy and conservative treatments were initiated following admission to the neonatal intensive care unit to manage infections and provide supportive care. Despite intensive medical interventions, the baby's condition remained critical due to the severity of HI and associated complications. Unfortunately, his health continued to deteriorate, and he succumbed to his condition after 14 days of life. His passing highlighted the challenges in managing this rare and severe genetic disorder, despite advances in neonatal care.

## 3 Discussion

The presentation of HI is typically immediate at birth, with infants displaying thick, rigid skin, deep fissures, and erythematous areas due to the formation of large, diamond‐shaped scales. The skin often appears cracked and tense, impairing the infant's ability to move and breathe effectively. Other manifestations include ear and eye deformities, as well as difficulty with thermoregulation and feeding [5].

HI results from mutations in the ABCA12 gene [1, 2, 3], which leads to defective lipid transport in the epidermal cells, causing defective barrier function [1, 4]. This defect leads to the excessive accumulation of keratinocytes, which form thick, adherent plaques that impair normal skin shedding [6].

The management of HI is primarily supportive, with the goal of preventing infections, maintaining hydration, and managing pain [6, 7]. Early interventions include the use of emollients and keratolytic agents to soften the scales and encourage their removal. Intravenous fluids and electrolytes may be required to correct imbalances caused by skin barrier dysfunction [8]. Antibiotic treatment is often necessary to prevent or treat infections, and respiratory support may be needed to ensure adequate oxygenation. Given the severity of the condition, early multidisciplinary care is crucial to improving outcomes. The involvement of dermatologists, neonatologists, physical therapy specialists, an orthopedic team, plastic surgeons, and pediatric specialists is key for providing comprehensive care [9].

Advances in neonatal intensive care have improved the survival rate of infants with HI. Many infants, once considered unlikely to survive beyond the first few days, can now live into childhood with appropriate care [10]. Long‐term management focuses on skin care, infection prevention, and addressing any systemic complications [5, 6].

Although life expectancy has improved, patients often face lifelong dermatological and psychosocial challenges.

## 4 Conclusion and Results

HI remains a challenging condition for both patients and clinicians, requiring early recognition and a multidisciplinary management approach. Advances in neonatal care have significantly improved survival rates, but the condition requires ongoing management throughout life. Early intervention, particularly in the neonatal period, is critical to reducing morbidity and mortality. Genetic counseling and prenatal diagnosis may be useful for families with a known risk of the condition.

## Author Contributions

**Shoaib Akhtar:** conceptualization, supervision, validation, visualization, writing – original draft, writing – review and editing. **Adeel Anwaar:** project administration, supervision, validation, visualization, writing – original draft, writing – review and editing. **Inam Ul Haq:** data curation, visualization, writing – original draft, writing – review and editing. **Hazaq Mukhtar:** visualization, writing – original draft, writing – review and editing. **Sabir Jamal:** writing – original draft, writing – review and editing. **Muhammad Muzammil:** writing – original draft, writing – review and editing. **Aymar Akilimali:** validation, visualization, writing – original draft.

## Ethics Statement

This is a case report utilizing anonymized patient information and so was classified as exempt from review from the institutional review board.

## Consent

A written informed consent was obtained from the patient based on the journal's policies.

## Conflicts of Interest

The authors declare no conflicts of interest.

## Data Availability

The authors have nothing to report.
